# Detection and quantification of a focal fat deposition in a liver undergoing multiple operations for neuroendocrine tumor disease using attenuation imaging: a case report

**DOI:** 10.1186/s13256-022-03723-x

**Published:** 2023-01-17

**Authors:** Elisabeth Miller, Wolfgang Kratzer, Angelika Kestler

**Affiliations:** Department of Internal Medicine I, University Hospital Ulm, Albert-Einstein-Allee 23, 89081 Ulm, Germany

**Keywords:** Attenuation imaging, Neuroendocrine tumor, Focal fat deposition, Case report

## Abstract

**Background:**

In patients with history of malignancy, new-onset liver lesions often present diagnostic challenges. We present the case of a patient with history of neuroendocrine tumor and new-onset echo-rich hepatic lesion, in whom attenuation imaging helped to make the diagnosis. Attenuation imaging is an ultrasound-based technique that allows for the quantification of hepatic fat content on the basis of a measurement of sound attenuation.

**Case presentation:**

We present the case of a 62-year-old Caucasian female patient who underwent pylorus-preserving pancreaticoduodenectomy Whipple surgery in 2004 for histologically well-differentiated neuroendocrine tumor with a proliferation rate of 3% of the pancreatic head. During the course, single liver metastases were resected in 2009, 2010, and 2013. In 2019, hemihepatectomy was performed when two liver metastases recurred. The liver metastases each showed a proliferation rate of 10% with vigorous expression of chromogranin A, synaptophysin, and somatostatin. The most recent follow-up examinations showed a normal chromogranin A value and the patient reported a good general condition. However, sonography revealed a blurred, echoic lesion in the liver. On contrast-enhanced sonography, the lesion showed identical behavior to the surrounding liver parenchyma. In the asymptomatic patient, liver biopsy did not seem to be indicated at the current time. Measurement of the attenuation coefficient by attenuation imaging showed a significantly higher measurement in the area of the echo-rich lesion than in the rest of the liver. The overall findings are consistent with focal fat deposition.

**Conclusions:**

Attenuation imaging appears to be useful in the evaluation of unclear echo-rich liver lesions. In particular, primary non-malignant-appearing liver lesions that are unremarkable on abdominal contrast-enhanced ultrasound can be more accurately assessed.

## Background

New-onset hepatic lesions present a diagnostic challenge in patients with previously diagnosed neuroendocrine tumors. In particular, atypical lesions that are not primarily malignancy-suspicious on B-scan ultrasound, such as cystic or echo-rich changes, often remain unexplained without liver biopsy. Due to good availability and risk-free and cost-effective procedures as well as sufficient sensitivity for metastasis detection in the liver, transabdominal ultrasound is considered a suitable method to document marker lesions in neuroendocrine tumors (NET) during the course according to the national S2K guideline. To achieve an increase in accuracy, contrast-enhanced ultrasound can be performed. Attenuation imaging is a relatively new ultrasound-based technique that allows for conclusions to be drawn about hepatic fat content by measuring the attenuation coefficient. Attenuation imaging is currently used to diagnose diffuse fatty liver, but the measurement box can be freely placed by the examiner, which allows placement even on a (sufficiently large) focal lesion.

## Case presentation

In this report, we present the case of a 62-year-old Caucasian female patient who underwent pylorus-preserving pancreaticoduodenectomy Whipple (pp-Whipple) surgery in 2004 for histologically well-differentiated neuroendocrine tumor with a proliferation rate of 3% of the pancreatic head. Subsequently, multiple liver metastasis resections of segment VI/VII in 2009, an abscessed liver metastasis recurrence with diaphragmatic involvement in 2010, an atypical liver segment resection in segment VI in 2013, and finally a right hemihepatectomy in 2019 were required when two new liver metastases were discovered. Initial histology showed pancreatic neuroendocrine tumor (pNET) with a proliferation rate of 3%, and a proliferation rate of 10% with vigorous expression of chromogranin A, synaptophysin, and somatostatin was seen at each of the metastatic resections. The patient regularly presented for follow-up examinations since the initial diagnosis. Chromogranin A levels were normal since disease onset, and the patient most recently showed no B-symptomatology or symptoms of hormone-responsive neuroendocrine tumor and good exercise tolerance (ECOG 0).

Since pp-Whipple surgery, recurrent cholangitis occurred, which was well treatable with antibiotic therapy. For the first time, an echo-rich blurred lesion was detected in the liver 9 months ago. The lesion did not show increased vascularization and did not show signs of NET recurrence on B-mode ultrasound. At the time of the onset of the lesion, an elevated C-reactive protein (CRP) value of 95 mg/l was detectable. Therefore, antibiotic therapy was initiated if cholangitis was suspected, and cholestasis values were also slightly elevated. In the short-term control, the CRP value was again completely normal, however, the hepatic lesion progressed sonographically in size, so that a correlate of the cholangitis undergone was not assumed. Subjectively, the patient showed wellbeing. Contrast-enhanced sonography was performed for further clarification of the lesion. Here, the echo-rich lesion behaved like the surrounding liver tissue. No perfusion defects could be visualized on contrast-enhanced ultrasonography. Another possible hypothesis discussed was local iron accumulation or focal/zonal fat deposition. For medical and ethical reasons as well as the long and sometimes complicated postoperative severe disease course, the patient did not consent to a new invasive liver biopsy. For further clarification, attenuation imaging, which provides a quantitative measurement of the fat content of the liver, was performed. Attenuation imaging (ATI) is integrated into the Aplio i800 device from Canon Medical Systems and can be performed with the conventional i8CX1 transducer. ATI quantifies the sound attenuation of the ultrasound signal and takes advantage of the fact that ultrasound waves are attenuated by the tissue properties of the liver parenchyma with increasing penetration depth [1]. The ATI calculation involves several steps so that finally a signal intensity profile as a function of depth is obtained, reflecting the scattering and absorption of the tissue [2]. The attenuation coefficient (AC) can be estimated by dividing the slope of the signal intensity by the transmit frequency. Thus, the system receives the returning echo signal and eliminates the focus-dependent beam profile and compensated gain profile from the original received signal. Then the system calculates the adjusted sound intensity and estimates the attenuation coefficient [3]. The AC is given in decibels per centimeter per megahertz, and should correlate with the degree of steatosis, as a high percentage of intrahepatic fat attenuates the ultrasound signal more than a low percentage of fat [4]. The measurement was performed in the Steatosis i8CX1 preset according to the manufacturer’s instructions [5]. For the measurement of attenuation, the patient was asked to place her right arm behind her head in the supine position. As a conventional B-scan is displayed in the left part of the screen during ATI, it is possible to place the measurement box in the area of the unclear echo-rich zone by using the trackball. During a short pause in the patient’s breathing, five measurements were taken in the area of the unclear echo-rich zone and five measurements were taken in B-mode sonographically unremarkable liver areas. The determination of median, mean, and interquartile range was automatically performed. The reliability of the measurement was displayed at the bottom left of the screen. A value of *R*^2^ > 0.90 was sought. The average AC is displayed directly after the measurement.

Here, it was found that in the area of the echo-rich altered liver, significantly increased values of 0.75 dB/cm/MHz could be measured. In areas of the B-Mode sonographically normal liver, the values were in the normal range with an average of 0.63 dB/cm/MHz. This demonstrates that the method is able to quantify and detect zonal hyperlipidemia in unclear liver findings.

## Discussion

At the current time, there is no known published comparable case in which attenuation imaging has been studied in patients with focal or zonal fat deposition.

**Fig. 1:**
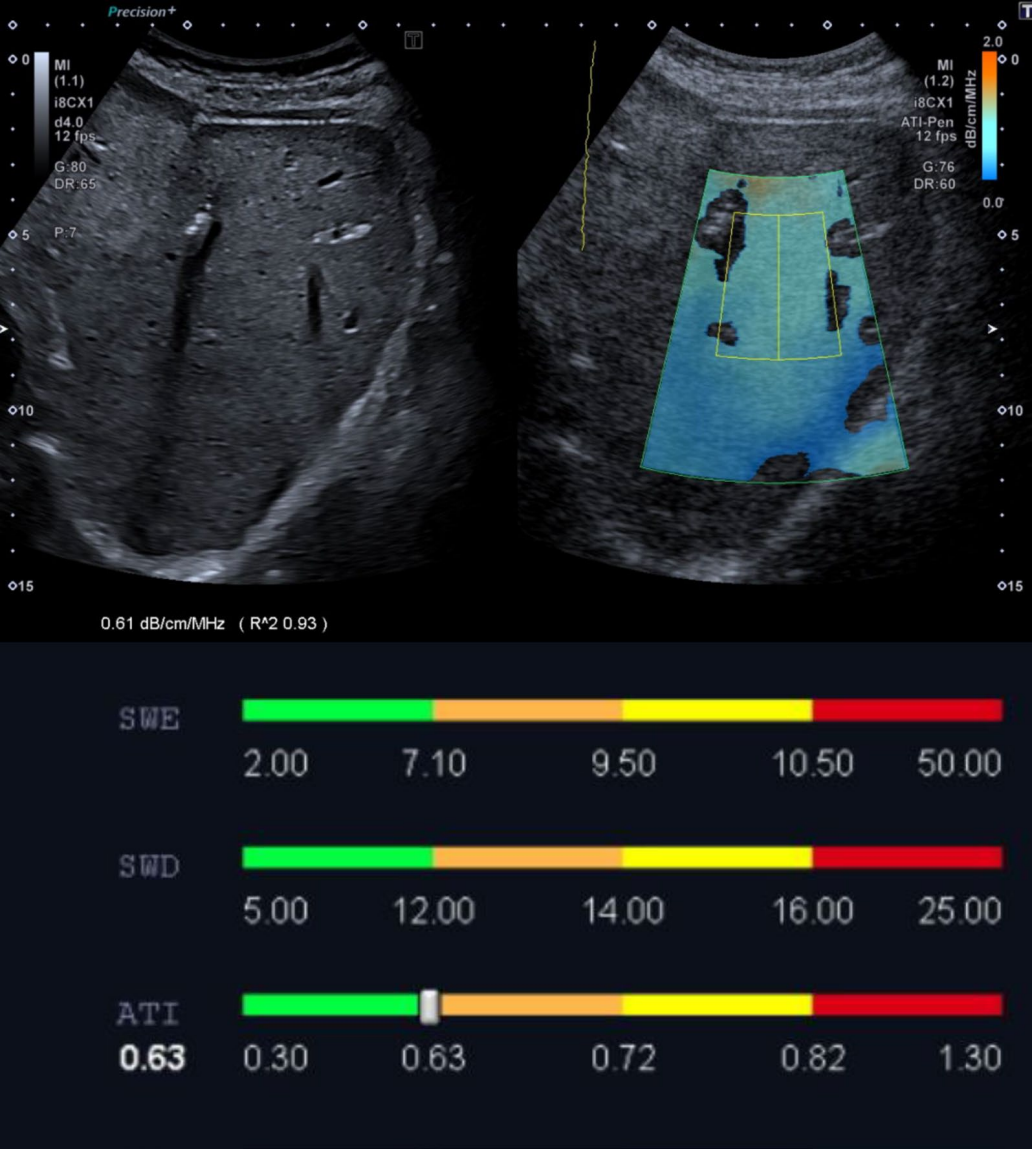
Attenuation imaging in the area of a B-scan sonographically inconspicuous liver region. The mean attenuation coefficient from five measurements is 0.63 dB/cm/MHZ

**Fig. 2:**
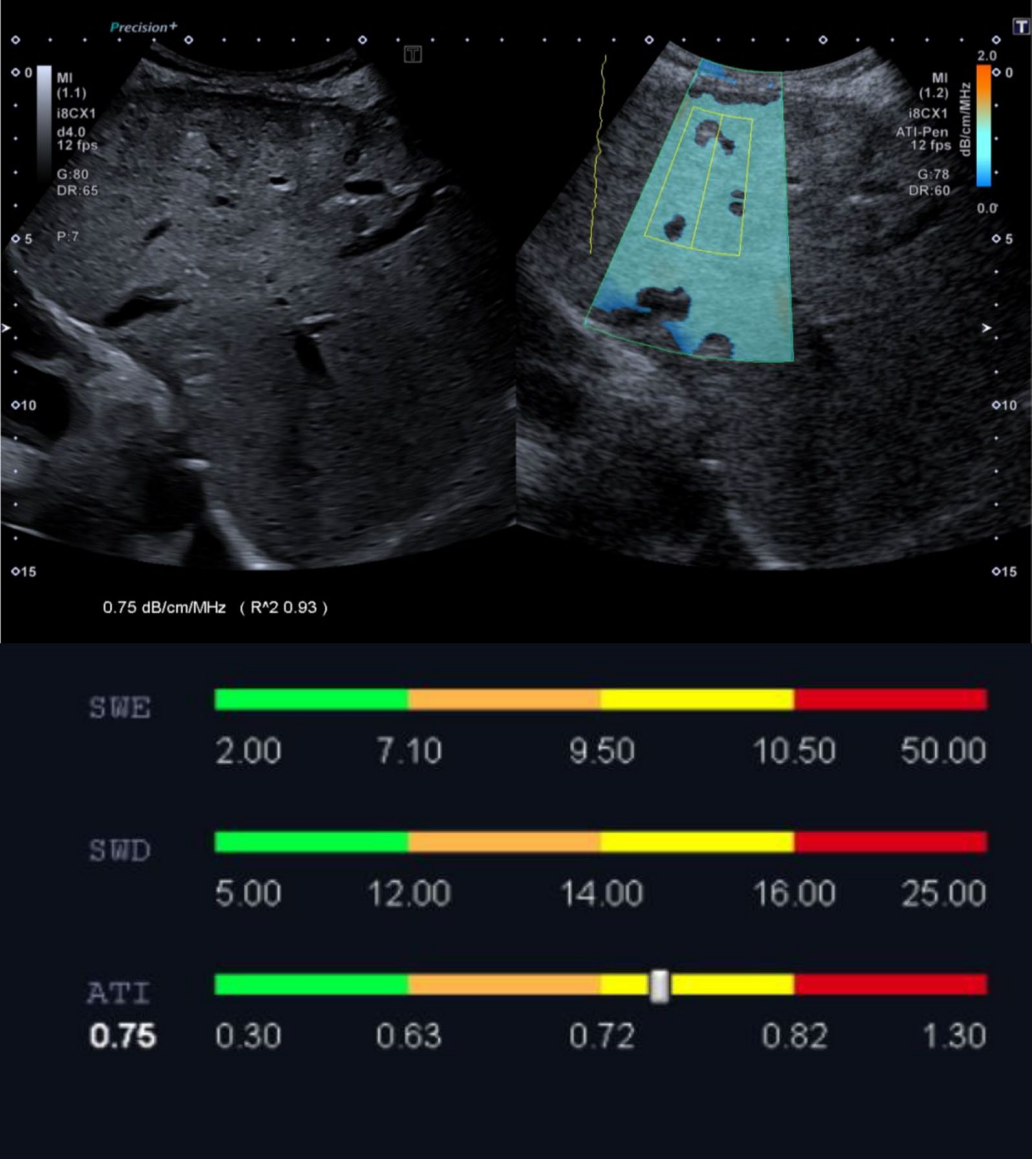
Attenuation imaging in the area of the zone appearing echo rich in the B-scan. The average attenuation coefficient from five measurements is 0.75 dB/cm/MHz

Previous studies have shown a statistically significant correlation between histologically diagnosed steatosis content and the attenuation coefficient [2, 4, 6, 7]. In the case described, the attenuation coefficient was significantly higher in the area of the zone that appeared echo rich on the B-scan than in the areas of the patient’s liver that appeared normal on the B-scan sonography. Considering the mean values from the five measurements, a clear difference between 0.63 dB/cm/MHz in the area of the inconspicuous liver areas (Fig. 1) and 0.75 dB/cm/MHz in the area of the echo-rich zone (Fig. 2) is noticeable.

If these values are placed in the context of previously published measurements, the impression arises that there is probably a high degree of fatty degeneration of the liver in the echo-rich zone. Bae *et al*. were able to show in a small patient collective that at an AC of > 0.745 dB/cm/MHz a high-grade steatosis with > 66% fatty hepatocytes can be detected with a sensitivity of 100% and a specificity of 82.4%. The area under the curve was 0.923 [6]. Tada *et al*. were able to detect fatty degeneration of > 66% of hepatocytes with a sensitivity of 100% and a specificity of 71.4% in *n* = 38 patients with non-alcoholic fatty liver disease (NAFLD) and a cut-off value of 0.74 dB/cm/MHz [2]. Another study also demonstrated good sensitivity and specificity of 96% and 74%, respectively, at an AC of > 0.72 dB/cm/MHz for the diagnosis of moderate–severe steatosis with a proportion of fatty hepatocytes of 33–66% and > 66%, respectively [7].

In that patient, a mean attenuation coefficient of 0.63 dB/cm/MHz could be measured in the area of the remaining liver. Bae *et al*. chose an AC of > 0.635 dB/cm/MHz as a cut-off value for S1 steatosis (> 5% fatty hepatocytes), with a sensitivity of 74.5% and a specificity of 77.4%. Furthermore, a high negative predictive value for the detection of clinically significant hepatic steatosis of more than 5% and more than 10%, respectively, was demonstrated. The degree of fibrosis and inflammation did not correlate with AC [6]. In Dioguardi Burgio *et al*. the mean attenuation coefficient in patients without steatosis hepatis in histopathological findings was 0.63 dB/cm/MHz [7]. Thus, presumably, there is no significant steatosis hepatis in the patient’s other liver areas.

Previous results of attenuation imaging, with correlation coefficients ranging from *r* = 0.91 to *r* = 0.98, suggest good intra- and interindividual reproducibility and, with *r* = 0.81, a high correlation of the results of ATI with the results of a magnetic resonance imaging proton density fat fraction (MRI-PDFF) examination as well [8].

Focal fat deposition is rarer than focal fatty sparing. The cause of fat deposition is not yet fully understood; various hypotheses are discussed, for example, increased storage of fat due to relative ischemia caused by reduced portal vein flow. Increased insulin levels in portal venous blood are also discussed. Focal fat deposition is often localized subcapsular. The localization of focal fat deposition and focal fatty sparing is similar, as both changes are presumably due to portal venous flow [9].

As a limitation, it must be mentioned that the presented case report does not allow for any conclusions about the assessment of the technique. Further prospective pilot studies are necessary to answer these questions.

## Conclusions

This case report demonstrates that attenuation imaging may help in the evaluation of echo-rich zonal changes in the liver. In particular, in cases where liver biopsy appears to be ethically unacceptable, attenuation imaging could help to establish the diagnosis.

## Data Availability

Not applicable.

## Abbreviations

NET: Neuroendocrine tumor
pNET: Pancreatic neuroendocrine tumor
ATI: Attenuation imaging
AC: Attenuation coefficient
MRI-PDFF: Magnetic resonance imaging proton density fat fraction
